# This is not normal: a call for HIV activism

**DOI:** 10.1002/jia2.70063

**Published:** 2025-11-27

**Authors:** Demetre C. Daskalakis

**Affiliations:** ^1^ Emory University Dr. Demetre Consulting LLC Atlanta Georgia USA

1

I have learned that successful public health programmes rely on the strength of three pillars: science, community and political will [1]. Each public health challenge requires a different degree of support from these pillars, but if any one of them is undermined, public health struggles to uphold its mission—to protect people's health. HIV serves as a compelling case study of this principle, both in the United States and globally.

The story begins with the pillar of community. AIDS in America was first identified in the early 1980s among gay men—a community that had only recently mobilized for its liberation. Faced with a deadly outbreak, the infrastructure of the gay rights movement shifted from fighting for civil rights to fighting for survival. This community demanded that science be prioritized and that political will be shown at a time when both were lacking in response to the emerging epidemic. The community stormed the US Food and Drug Administration and the US National Institute of Health, staged powerful symbolic protests, burned effigies in public and forged strong alliances with government and scientists in private. This surge of community activism not only facilitated scientific efforts, but also helped shape and accelerate them, resulting in steady progress and major breakthroughs driven by, and for, the community.

Because of this, progress against HIV has often outpaced changes in society over the past three decades. Antiretroviral medications transformed HIV into a chronic, manageable disease for those who could access treatment. Later, it was discovered that the same treatment preventing disease progression also stopped sexual transmission of the virus—giving rise to the concept “Undetectable = Untransmittable” [2]. Pre‐exposure prophylaxis moved quickly from clinical trial results to real‐world implementation, albeit unevenly. Long‐acting injectable treatments and preventatives have further strengthened the idea that, even without a vaccine, controlling the HIV epidemic is possible.

The pillar of political will has always played a crucial role in the response against HIV, carrying much of the weight and contributing significantly to its successes. Community members and scientists insisted that scientific advances be matched by programmes that respected affected populations and delivered these life‐saving interventions in ways that responded to community needs. HIV, seemingly immune from partisan politics, became a story of success—a “moonshot” achieved through the combined efforts of community, science and government. Three programmes created by US Republican administrations stand out as examples of what the United States accomplished both globally and domestically:

- The Ryan White AIDS Program was established in 1990 during the administration of President George H.W. Bush, following the death of Ryan White, a teenager who became a national symbol for HIV/AIDS advocacy after facing discrimination due to his diagnosis. The programme was designed to provide comprehensive care and support for people living with HIV/AIDS who lacked sufficient resources, marking a major federal commitment to fighting the epidemic [3].
- The President's Emergency Plan for AIDS Relief (PEPFAR) was launched in 2003 under President George W. Bush. This landmark initiative aimed to address the global HIV/AIDS crisis, particularly in the hardest‐hit countries, by providing funding and support for prevention, treatment and care programmes. Since its inception, PEPFAR has provided antiretroviral therapy to millions worldwide. As of 2024, it has supported lifesaving antiretroviral therapy (ART) for over 20 million men, women and children, making it one of the largest public health efforts dedicated to combating HIV/AIDS [4].
- The Ending the HIV Epidemic Program began under the first Trump Administration (2019) as a national initiative to reduce new HIV acquisitions in the United States. Its launch marked a renewed federal commitment to leveraging scientific advances, community engagement and political will to end the epidemic [5].

This period was, in many ways, a golden age for public health and science. But, as with all golden ages, challenges soon emerged. When political will wanes, the foundation is at risk of crumbling. When science is undermined by cultural battles and deprioritized by funders, the breakthroughs of previous decades can fade into memory. And when vital programmes are threatened with elimination by the very politicians who created them, both progress and people's health are put in jeopardy.

This is where we stand today. Both domestic and global HIV funding now face serious threats, caught in a storm of politics that intentionally disregards health equity. Current rhetoric labels gender identity as an ideology rather than a personal expression, prioritizes efforts to “end wokeness” over the goal of ending the HIV epidemic, misrepresents global HIV initiatives as wasteful “foreign aid,” and seeks to dismantle critical funding for the Ryan White Program, the US Centers for Disease Control and Prevention's Division of HIV Prevention and PEPFAR [6, 7, 8].

The US Federal budget is a moral document. What it does or does not fund reflects our values as a nation—we should value the programmes that prevent death and disability from HIV. It is that simple. Choosing harmful priorities will cost lives. The United State's HIV response has always depended on bipartisan support—now, that legacy is endangered. The current administration should be remembered for saving lives, not losing them.

Despite attacks on public health, the misuse of science and attempts to silence our community, we must remain resilient. We have overcome adversity before, united by solidarity and we will do so again. Our community's spirit is our guiding light, even when hope feels distant.

As efforts undermine our success, we must do all we can to protect the health of people living with or at risk of HIV. We must be aware that the destructive decisions and actions of governments seemingly unbothered by public health are not free from consequences on the lives of people. HIV rates could increase, AIDS diagnoses surge, disparities deepen and once empty wards fill again. This is not normal, but we must be prepared. Rather than pine away for the HIV exceptionalism of the past, we need to imagine a new HIV strategy that is more resilient and resistant to the opportunistic attacks of today that are weakening the security of our globe. Focusing on communities and people and not a virus or one's HIV status will be key—a status‐neutral vision is no longer just a good idea, it is the answer to this existential threat that will protect our mission from misguided ideology [9].

We must also recognize the trauma of this moment. This is not normal, and we should not accept it as such. Self‐care is essential in our HIV workforce. As Audre Lorde said, “Caring for myself is not self‐indulgence, it is self‐preservation, and that is an act of political warfare” [10].

By sustaining ourselves, we sustain our community and keep alive the commitment to the health of the people we serve and the science we forge that will, one day, help us rebuild the political will needed to defeat HIV.

## COMPETING INTERESTS

The author declares no competing interests.

## AUTHOR CONTRIBUTIONS

DCD wrote and review the article.

## Data Availability

Data sharing is not applicable to this article as no new data were created or analysed in this study.
